# Burden of SARS-CoV-2 infection and severe illness in South Africa (March 2020–August 2022): a synthesis of epidemiological data

**DOI:** 10.1136/bmjph-2024-002174

**Published:** 2025-11-21

**Authors:** Larisse Bolton, Stefano Tempia, Sibongile Walaza, Waasila Jassat, Kaiyuan Sun, Debbie Bradshaw, Rob Dorrington, Jackie Kleynhans, Neil Martenson, Anne von Gottberg, Nicole Wolter, Juliet R C Pulliam, Cheryl Cohen

**Affiliations:** 1 South African Centre for Epidemiological Modelling and Analysis (SACEMA), School for Data Science and Computational Thinking, University of Stellenbosch, Stellenbosch, Western Cape, South Africa; 2 Centre for Respiratory Diseases and Meningitis, National Institute for Communicable Diseases of the National Health Laboratory Service, Johannesburg, South Africa; 3 School of Public Health, University of the Witwatersrand, Johannesburg, South Africa; 4 Division of International Epidemiology and Population Studies, Fogarty International Center, National Institutes of Health, Bethesda, Maryland, USA; 5 Burden of Disease Unit, South African Medical Research Council, Cape Town, South Africa; 6 Department of Family Medicine and Public Health, University of Cape Town, Rondebosch, South Africa; 7 Centre for Actuarial Research (CARe), University of Cape Town, Rondebosch, South Africa; 8 Perinatal HIV Research Unit, University of the Witwatersrand, Johannesburg, South Africa; 9 School of Pathology, University of the Witwatersrand, Johannesburg, South Africa

**Keywords:** SARS-CoV-2, Public Health, Disease Notification

## Abstract

**Introduction:**

Data on the burden of SARS-CoV-2 infections by age group and for different severity levels are lacking. We estimated the South African SARS-CoV-2 disease burden and severity, describing changes in the shape of the disease burden pyramid with successive waves.

**Methods:**

We estimated SARS-CoV-2 medically and non-medically attended illness stratified by severity (mild, severe non-fatal and fatal) during the initial five waves, spanning 1 March 2020 through 13 August 2022. We used individual-level national surveillance, healthcare utilisation and serosurvey data to estimate wave-specific hospitalisation-fatality (HFR) and infection-fatality ratios. We estimated wave-specific incidence rates per 100 000 population with 95% CIs derived from bootstrapping the individual-level data.

**Results:**

On 13 August 2022, the estimated cumulative number of SARS-CoV-2 infections in South Africa was 104.6 million, of which 399 900 (0.38%) were severe non-fatal and 258 800-289 000 (0.25 - 0.28%) fatal. 29% of severe non-fatal illness and 55 - 60% of deaths occurred outside the hospital. The highest burden of severe and fatal illness was during the Delta wave (wave 3), and the HFR across the initial three waves was similar (range 31–34%). Although there were more infections during the Omicron BA.1 wave (wave 4), there was a substantial reduction in HFR (14%). Successive waves saw a reduction in the rate of increase in mortality and hospitalisations with increasing age.

**Conclusions:**

The substantial South African national burden of SARS-CoV-2 for the initial five waves contradicts the belief of minimal impact in Africa. A high proportion of severe non-fatal and fatal illness occurred outside of the hospital, highlighting the importance of studies of health-seeking and vital registration systems to document the full burden of illness. The highest burden of severe illness and death was in the Delta wave. Following the Omicron emergence, severe illness reduced, and with successive waves, proportionately more children were infected and became ill, suggesting a transition to a more endemic pattern.

**WHAT IS ALREADY KNOWN ON THIS TOPIC**

- As SARS-CoV-2 spread globally, initial assumptions painted a bleak picture for Africa due to its existing challenges in healthcare service delivery, multimorbidity, poverty and lack of resources needed to fight the infection. The number of cases and deaths reported during the pandemic seemed to contradict these initial assumptions. South Africa recorded over 4 million laboratory-confirmed cases of COVID-19 during the first 3 years of the pandemic. However, it is estimated that only a 10th of the cases were diagnosed. With the lack of testing, inconsistent healthcare-seeking behaviour, changes in attack, reinfection and symptomatic rates, the true burden of SARS-CoV-2 across the different age groups and severity levels was largely unknown. Although real-time epidemiological data were crucial for informing intervention strategies throughout the pandemic, it is now essential to quantify and describe the evolution of the epidemiology over successive pandemic waves as more information was made available.

**WHAT THIS STUDY ADDS**

- We found a high burden of severe illness and death in the first three waves of SARS-CoV-2, peaking in the third (Delta) wave. The emergence of the Omicron BA.1 variant was associated with very high rates of infection but substantial reductions in disease severity. Incidence of severe illness and hospitalisation fatality generally increased with increasing age. Successive waves saw a reduction in the rate of the increase in mortality with increasing age and increases in hospitalisation-fatality ratios in children below 5 years of age, suggesting a shift from the epidemic state to a J-shaped distribution in mortality, typical of seasonal respiratory viruses. Notably, a high proportion of severe illness (29%) and death (55-60%) occurred outside the hospital.

**HOW THIS STUDY MIGHT AFFECT RESEARCH, PRACTICE OR POLICY**

- Our study provides insights into the changes in patterns of infection and disease following the introduction of a novel pathogen into a susceptible population, which may be useful for future pandemic planning. The high proportion of undiagnosed and unreported illness and the high proportion of severe illness and death occurring outside of the hospital suggest that strengthening access to diagnosis and care is needed in our setting.

## Introduction

 As of the end of August 2023, confirmed numbers of COVID-19 cases and deaths worldwide exceeded 770 million and 6.9 million, respectively.^1^ South Africa is an upper middle-income country in Sub-Saharan Africa with a total population of more than 60 million individuals, 28% of whom are aged below 15 years.^2 3^ HIV is an important comorbidity for respiratory illness in South Africa, with a prevalence of 12.6% in 2022.^3^ More than 4 million laboratory-confirmed cases of COVID-19 were identified in South Africa from the first confirmed case in early March 2020 until 25 March 2023,^4^ cumulatively comprising almost half (42.7%) of the total confirmed COVID-19 cases in Africa.^5^ SARS-CoV-2 testing capacity in Africa is limited, and in South Africa, less than 10% of all SARS-CoV-2 cases during the first three waves are estimated to have been diagnosed.^6 7^ South Africa’s vaccination roll-out was initiated among healthcare workers in February 2021 and extended to the elderly and adolescents in May and October 2021, respectively.^8^ Vaccination coverage within South Africa reached 27% by 12 January 2022.^9^

From 5 March 2020 through 31 August 2022, South Africa experienced five waves of SARS-CoV-2, each dominated by a different variant (D614G, Beta, Delta, Omicron BA.1/2 and Omicron BA.4/5, respectively).^10^ As the virus becomes endemic with successive waves of infection, a rise in population SARS-CoV-2 immunity induced by natural infection and vaccination can lead to changes in the epidemiology of disease.^11^,^13^ These changes may include reductions in disease severity, as well as changes in the overall burden and age distributions of infections, illnesses and deaths.^12 13^ Furthermore, different emerging SARS-CoV-2 variants may be associated with immune evasion and variation in virulence, infectiousness and/or transmissibility, which can affect rates of infection and reinfection as well as variants’ intrinsic severity of illness.^14^,^16^

South Africa’s government-funded^17^ public healthcare sector is the primary provider of healthcare for the majority of the population,^18^ yet resources are limited.^19^ Although public clinics, hospitals or other institutions are the primary point of healthcare access for 72% of the population, healthcare-seeking behaviour varies.^18^ Considering the lack of testing as well as variable healthcare-seeking behaviour and changes in the symptomatic proportion, attack rates and reinfection frequency across the five SARS-CoV-2 waves, the shape of the disease burden pyramid is largely unknown.

Data on the burden of SARS-CoV-2 in successive waves across different levels of severity are limited globally. This is particularly important in Africa, where resources are limited and official statistics mistakenly suggest that the burden of cases and mortality was substantially lower than in other regions.^5^ We aimed to estimate the burden of SARS-CoV-2 infection and severe disease in South Africa from March 2020 through August 2022, and to describe the variation in levels of severity in infected individuals (ie, the disease burden pyramid) across the first five pandemic waves. At the start of the pandemic, it was critical to have realistic epidemiological data to model the impact of interventions. Early models were used to support South African policy makers, updating stakeholders and the South African public throughout the course of the pandemic.^20^ In this study, we, however, use more data sources than were available in real time.

## Materials and methods

### Conceptual outline of burden strata with data sources

#### Strata definitions

**Figure 1:**
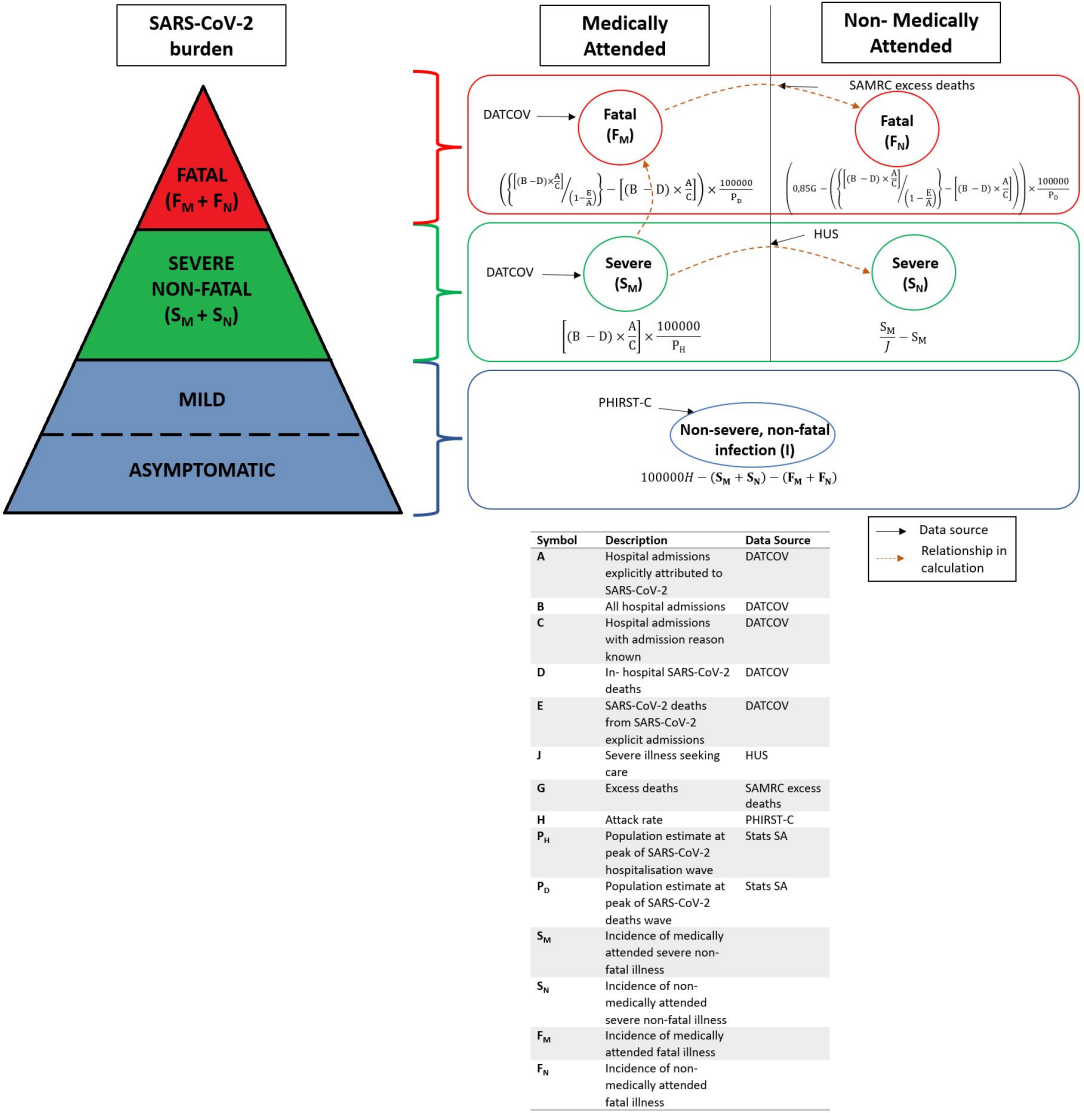
A summary illustration of the burden pyramid and related disease severity strata by medical attendance, with data inputs and relationships between strata. For details on calculations, refer to the Severe disease and death overall section. DATCOV, national surveillance programme for SARS-CoV-2 hospitalisations^8^; HUS, healthcare utilisation survey^24^; SAMRC excess deaths, South African Medical Research Council estimates of excess deaths^22^; PHIRST-C, Prospective Household study of SARS-CoV-2, Influenza and Respiratory Syncytial virus community burden, Transmission dynamics and viral interaction in South Africa—Coronavirus disease 2019.^25^

The SARS-CoV-2 burden comprises both asymptomatic infection (no clinical manifestation of disease) and symptomatic illness. We estimated the burden in three mutually exclusive severity strata: non-severe infection (asymptomatic infection and mild or moderate illness), severe non-fatal illness and fatal illness.^21^ Severe non-fatal and fatal illness was further stratified into medically and non-medically attended illnesses (figure 1). Individuals with severe illness who were admitted to the hospital or individuals who died in hospital were considered medically attended. Individuals with severe illness who were not hospitalised and those who died out of hospital were considered non-medically attended.^21^

#### Data sources

1. *Individual-level SARS-CoV-2 public and private hospital admissions and subsequent in-hospital deaths from the DATCOV national surveillance programme for SARS-CoV-2 hospitalisations*.^8^ DATCOV includes data on COVID-19 hospitalisations from all private and public healthcare facilities in South Africa. Hospitalised patients comprised individuals with a positive SARS-CoV-2 test, irrespective of whether hospitalisation was attributable to SARS-CoV-2 or not. Reporting of all laboratory-confirmed cases was legally mandated. DATCOV variables included demographic details such as age and sex, admission information such as the date and reason for admission, and patient outcome such as death or discharge.
2. *South African Medical Research Council (SAMRC) estimates of excess deaths by sex and 5-year age bands*.^22^ The SAMRC, in partnership with the University of Cape Town, adapted the existing annual Rapid Mortality Surveillance process to produce a near real-time (weekly) system for following and observing COVID-19 excess deaths. Weekly updates to the deaths recorded on the National Population Register, together with the classification of deaths due to natural or unnatural causes, were obtained and adjusted for both under and late registration of deaths. Excess SARS-CoV-2-associated deaths are estimated by comparing observed to expected deaths derived from a negative binomial regression model of deaths in pre-COVID-19 years, described in detail elsewhere.^23^ While we attempted to account for the proportion of deaths attributable to SARS-CoV-2, robust data on this are lacking, and it is possible that this varied over the pandemic.
3. *Healthcare utilisation surveys (HUSs) conducted in three communities in three provinces*.^24^ From November 2020 through April 2021, fieldworkers enrolled 23 003 individuals from 5804 randomly selected households. All individuals reporting severe respiratory illness (SRI) since the start of the SARS-CoV-2 pandemic were asked about healthcare-seeking behaviour, including the proportion of these seeking care at healthcare facilities.
4. *Age-stratified infection attack rates by SARS-CoV-2 wave obtained from the Prospective Household study of SARS-CoV-2, Influenza and Respiratory Syncytial virus community burden, Transmission dynamics and viral interaction in South Africa–Coronavirus disease 2019 (PHIRST-C) cohort*.^25^ This cohort comprised 1200 participants randomly selected from two communities in two provinces, including 643 participants from a rural site in Agincourt (Mpumalanga Province, South Africa) and 557 participants from an urban site in Jouberton (North-West Province, South Africa). 10 consecutive serum specimens were obtained from each participant between July 2020 and April 2022 and tested for anti-SARS-CoV-2 antibodies.^6 7 14 25^ The estimated age-stratified infection attack rates from the urban site were employed in calculations, because these were thought to be more representative of South Africa as a whole, since the majority of South Africans live in urban areas.^26^ The infection attack rate was defined as the number of new infections divided by the population at risk over a defined period.^27^
5. *Midyear population estimates*, unstratified and stratified in 5-year age bands and sex strata, provided by the government agency, Statistics South Africa.^28 29^

### Definitions

#### Wave

The total number of SARS-CoV-2 hospitalisations and in-hospital deaths was calculated by epidemiological year (year) and epidemiological week (epiweek). The peaks (maximum SARS-CoV-2 hospitalisations and in-hospital deaths), as well as the nadirs (minimum hospitalisations and in-hospital deaths between these peaks), were then determined. For both hospitalisations and in-hospital deaths, the first wave was defined to extend from the start of the epiweek associated with the start of the pandemic in South Africa to the end of the epiweek of the nadir after this peak. The subsequent waves were defined to extend from the start of the epiweek following the nadir prior to the peak till the end of the epiweek of the nadir following the peak (online supplemental figure 1, online supplemental table 1). However, to achieve agreement between the in-hospital and SAMRC excess deaths wave definitions, an epiweek was included at the end of wave 2 and the start of wave 3. Wave 5 was truncated at epiweek 32 of 2022. The SARS-CoV-2 hospitalisations and in-hospital deaths wave definitions were employed for the respective hospitalisation and in-hospital fatalities stratum estimates.

#### Country population at wave peak

Weekly population estimates were obtained by linear interpolation of midyear population estimates.

### Estimation approach

All analyses and calculations were carried out in RStudio 2023.06.0+421 for Windows 10 Enterprise version 22H2^30^ (except bootstrapping; RStudio version 2025.05.1+513 for Windows 10 Enterprise version 1909), running R Statistical software V.4.2.3.^31^ The burden estimation approach is outlined hereafter. For a detailed description of the burden estimation approach, refer to online supplemental file 2. Calculations for each stratum were implemented on an age-stratified and per-wave basis unless otherwise noted. Furthermore, national burden estimates were subsequently obtained from age-stratified figures (refer to online supplemental files 1 and 2 for details). The burden pyramid and related disease severity strata with data inputs and relationships between strata are depicted in figure 1.

#### Severe non-fatal and fatal illness

##### Medically attended severe non-fatal illness (incidence of hospitalisation)

Using the SARS-CoV-2 hospitalisation wave definitions within DATCOV, the medically attended severe non-fatal illness is defined as the difference between all hospital admissions and total in-hospital deaths multiplied by the proportion of hospital admissions explicitly attributed to SARS-CoV-2 of all hospital admissions with admission reasons known. The incidence rate per 100 000 (*S*_*M*_) is obtained by dividing by the respective national population estimate at the peak of the SARS-CoV-2 hospitalisation wave followed by multiplying by 100 000.

##### Medically attended fatal illness (incidence of in-hospital death)

The medically attended fatal illness incidence rate per 100 000 (*F_M_*) was determined as follows: the adjusted number of total hospitalisations was calculated by dividing the adjusted number of SARS-CoV-2 hospitalisations by the proportion of hospitalisations that did not result in death, that is, 1—HFR, subtracting out the adjusted SARS-CoV-2 hospitalisations and dividing by the respective national population estimate at the peak of the SARS-CoV-2 in-hospital deaths wave, finally multiplying by 100 000.

##### Non-medically attended severe non-fatal illness

The incidence of non-medically attended severe non-fatal illness (*S*_*N*_) is defined as the difference between the incidence of hospitalisation (*S*_*M*_) divided by the average proportion of severe illness seeking care from the HUS and the incidence of hospitalisation (*S*_*M*_). The HUS data were only available for three provinces for the first two waves. However, due to the lack of HUS data for the subsequent waves, we assumed the average proportion of severe illness seeking care from the HUS remains unchanged irrespective of age strata or wave.

##### Non-medically attended fatal illness (incidence of out-of-hospital deaths)

We assumed that 85% of excess deaths were attributable to SARS-CoV-2 based on published data.^32^ The incidence of out-of-hospital deaths per 100 000 (*F*_*N*_) was then calculated as the SARS-CoV-2 attributable excess deaths less the adjusted SARS-CoV-2 in-hospital deaths, divided by the respective national population estimates at the peak of the SARS-CoV-2 death waves times 100 000. For the age-specific estimates, age groups for which in-hospital deaths exceeded the attributable excess deaths, the out-of-hospital deaths estimate was set to zero, that is, the SARS-CoV-2 attributable excess deaths were assumed equal to the in-hospital deaths.

### Non-severe, non-fatal illness (ie, moderate, mild and asymptomatic illness)

The non-severe, non-fatal infection incidence (I) was obtained by multiplying the wave and age-specific attack rates from PHIRST-C (refer to Data sources section (4)) by 100 000 and subtracting out the medically and non-medically attended severe non-fatal and fatal illness, since disease severity strata are assumed mutually exclusive.

### Estimation of uncertainty

CIs surrounding the incidence estimates were obtained from bootstrapping the individual-level data. That is, the wave-specific admission and in-hospital death data from DATCOV were each randomly sampled with replacement 1000 times, stratified by wave. The average proportion of severe illness seeking care from the HUS was determined using a random binomial distribution with a probability of success equal to the proportion of people seeking care in the HUS and the number of trials equal to the sample size. For each run, the number of successes obtained by the random binomial distribution was divided by the sample size to obtain the proportions of people seeking care. It was assumed that the health-seeking behaviour was consistent across age groups due to a lack of HUS data to proceed otherwise. Similarly, the age-specific attack rates were obtained from the random binomial distribution with a probability of success defined as the true attack rate from PHIRST-C and the number of trials equal to the sample size. For each run, the number of successes was once again divided by the sample size. The resulting simulated attack rate was then compared with a threshold criterion to prevent the calculated infection incidence from being negative. That is, should the generated random attack rate times 100 000 not be greater than the maximum of the sum of the 95% CI upper bounds of the total incidence of severe non-fatal illness and total incidence of fatal illness, we continue to draw random attack rates until the threshold is exceeded. 95% CIs were estimated as the 2.5% and 97.5% quantiles of the respective bootstrapped/simulated estimates. No CIs were generated for the incidence of out-of-hospital deaths and total incidence of death because we are unable to quantify the uncertainty associated with the proportion of total deaths due to SARS-CoV-2. CIs for the incidence of non-severe non-fatal and non-fatal illness, infection-fatality ratio (IFR) and severe respiratory infection-fatality ratio (SRIFR) are solely derived from the uncertainty around the infection attack rates and incidence of severe non-fatal non-medically and medically attended illness. We have, however, conducted an additional sensitivity analysis on varying proportions of excess deaths attributable to SARS-CoV-2 to provide a plausibility range (refer to online supplemental table S3 and onlinesupplemental files 3^7^). Detailed computational outputs from the analyses are provided in online supplemental materials.

### Patient and public involvement

The study involved a synthesis of retrospective data, and thus it was not possible to involve patients or the public in the design, conduct, reporting or dissemination plans of this research.

## Results

### Overall

Overall, across the first five waves of SARS-CoV-2 in South Africa, from 1 March 2020 through 13 August 2022, we estimate that there were 104.6 million SARS-CoV-2 infection episodes, of which 399 900 (0.38%) were severe non-fatal illness episodes and 258 800–289 000 (0.25–0.28%) deaths out of a population of approximately 300 million individuals under observation over the period (online supplemental figure 1). 29% of severe non-fatal illness and 55–60% of deaths occurred outside of the hospital.

### Severe disease and death overall

**Figure 2:**
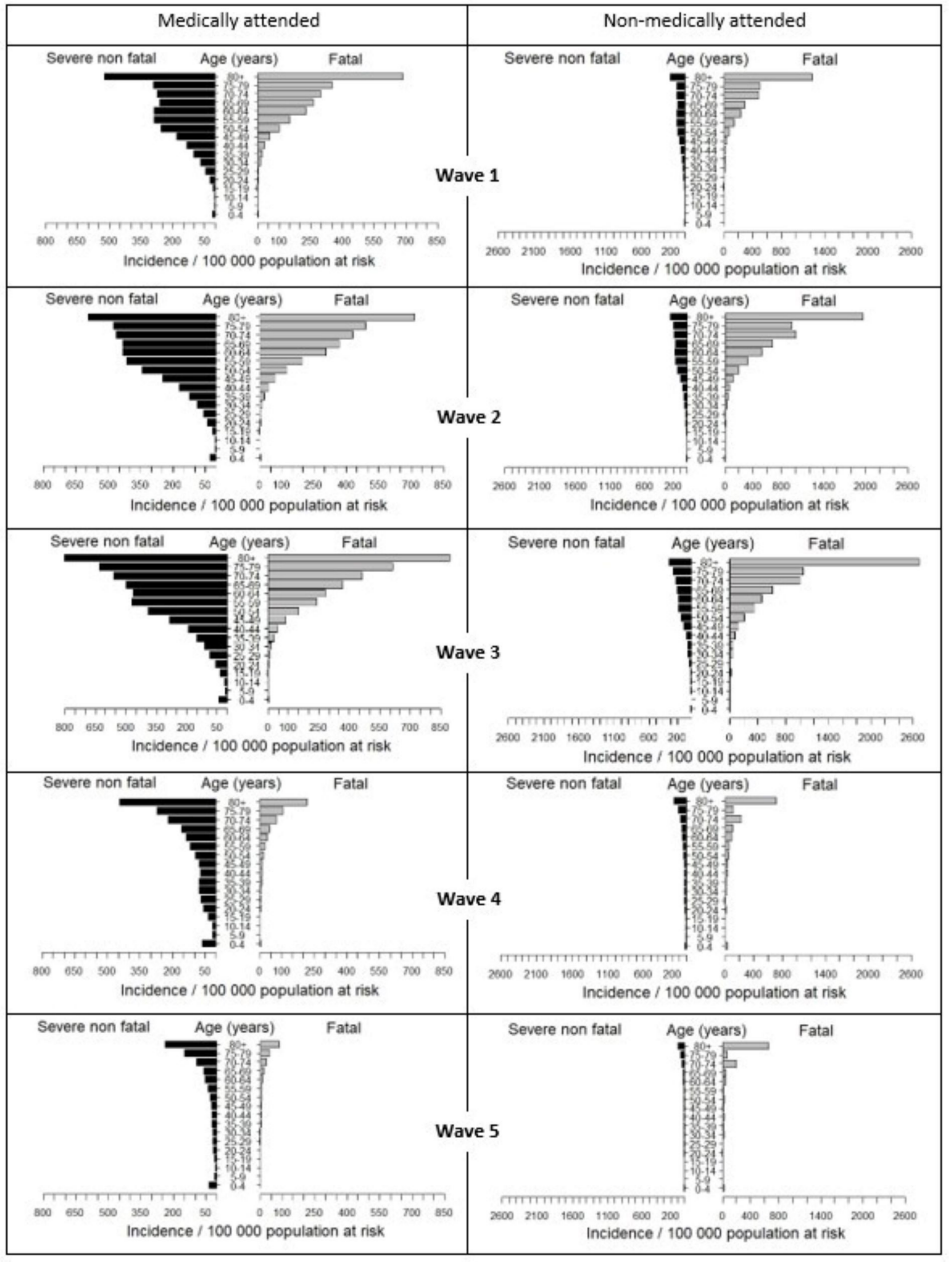
Age-specific incidence of severe and fatal illness by age for the first five SARS-CoV-2 waves in South Africa by medical attendance from 01 March 2020 through 13 August 2022.

Incidence of both medically- and non-medically attended severe non-fatal and fatal illness increased from wave 1 to 3, with the highest incidence occurring during the Delta wave (wave 3) (figure 2, table 1). The incidence of death, both medically- and non-medically attended, also increased across the first three waves, peaking during the Delta (third) wave, with 69.2 (95% CI 67.6 to 70.8) in-hospital and 87.1 out-of-hospital deaths per 100 000 population, respectively. Thereafter, severe illness and death incidences decreased progressively in waves 4 and 5. The HFR was approximately 32% in the first three waves, decreasing slightly from wave 1 to 3 (from 34.1% (95% CI 33.2% to 35.1%) to 30.6% (95% CI 30.1% to 31.1%)), but then dropped substantially in wave 4 and 5 (to 14.4% (95% CI 14.0% to 14.9%)). In contrast, the out-of-hospital SRIFR showed no consistent trend, ranging from 36.9% (95% CI 28.4% to 46.1%) to 65.3% (95% CI 56.9% to 73.7%) across all five waves (table 2).

**Table 1:** Wave-specific incidence of severe non-fatal and fatal SARS-CoV-2 infections in South Africa by medical attendance per 100 000 population at risk from 01 March 2020 through 13 August 2022

Wave number/ dominant variant	Medically attended		Non-medically attended		Total		
	Incidence/100 000 population **(**95% CI**)**		Incidence/100 000 population (95% CI)		Incidence/100 000 population (95% CI)		
	Severe non-fatal	Fatal	Severe non-fatal	Fatal*	Severe non-fatal	Fatal*	Non-severe non-fatal and non-fatal†
1/ D614G	91.3 (90.7 to 91.9)	47.3 (45.6 to 49.2)	36.7 (24.5 to 52.2)	20.9	128.0 (116.1 to 143.6)	68.2	25 215.1 (18 421.8 to 27 884.6)
2/ Beta	129.1 (128.6 to 129.7)	61.3 (59.9 to 62.8)	51.9 (34.4 to 73.8)	81.7	181.0 (164.0 to 202.8)	143.0	23 829.5 (20 516.9 to 29 106.6)
3/ Delta	156.6 (155.9 to 157.2)	69.2 (67.6 to 70.8)	62.9 (41.8 to 89.6)	87.1	219.5 (199.2 to 246.3)	156.2	33 991.5 (28 477.3 to 37 441.5)
4/ Omicron BA.1/2	72.7 (72.2 to 73.2)	12.2 (11.8 to 12.7)	29.2 (19.4 to 41.6)	27.3	101.9 (92.3 to 114.3)	39.5	56 612.3 (53 114.7 to 62 679.4)
5/ Omicron BA.4/5	26.7 (26.3 to 27.0)	4.2 (3.9 to 4.5)	10.7 (7.1 to 15.3)	20.2	37.4 (34.0 to 42.0)	24.4	33 324.7 (31 033.6 to 40 980.1)

* CIs are not provided for out-of-hospital deaths or total incidence of deaths because we are unable to quantify the uncertainty associated with the proportion of excess deaths attributable to SARS-CoV-2 infection (assumed to be 0.85).

† Non-severe non-fatal and non-fatal = (infection attack rate × 100 000) − total incidence of severe non-fatal − total incidence of fatal.

**Table 2:** Wave-specific total numbers of SARS-CoV-2 infections, hospitalisations and deaths and hospitalisation-,severe respiratory illness- and infection-fatality ratio in South Africa from 01 March 2020 through 13 August 2022.

Wave number	Number of infections (millions)	Number of hospitalisations (millions)	Number of in-hospital deaths (millions)	Number of out-of-hospital deaths (millions)	Number of severe non-fatal illness (non-hospitalised) (millions)	HFR* (%) (95% CI)	SRIFR† (%) (95% CI)	IFR‡ (%) (95% CI)
1	15.09	0.054	0.028	0.013	0.022	34.1 (33.2 to 35.1)	36.9 (28.4 to 46.1)	0.27 (0.24 to 0.37)
2	14.42	0.077	0.037	0.049	0.031	32.2 (31.7 to 32.7)	61.1 (52.5 to 70.2)	0.59 (0.49 to 0.69)
3	20.62	0.094	0.042	0.052	0.038	30.6 (30.1 to 31.1)	58.0 (49.3 to 67.5)	0.45 (0.41 to 0.54)
4	34.22	0.044	0.0074	0.017	0.018	14.4 (14.0 to 14.9)	48.3 (39.5 to 58.3)	0.070 (0.063 to 0.074)
5	20.22	0.016	0.0025	0.012	0.0065	13.5 (12.6 to 14.6)	65.3 (56.9 to 73.7)	0.073 (0.059 to 0.078)

* HFR (%) = (number of SARS-CoV-2 in-hospital deaths/(number of SARS-CoV-2 hospital admissions)) × 100.

† SRIFR (%) = (incidence of fatal non-medically attended COVID-19/(incidence of severe non-fatal non-medically attended COVID-19 + incidence of fatal non-medically attended COVID-19)) × 100.

‡ IFR (%) = (total incidence of fatalities/(infection attack rate × 100 000)) × 100.

HFR, hospitalisation-fatality ratio; IFR, infection-fatality ratio; SRI, severe respiratory illness; SRIFR, severe respiratory infection-fatality ratio.

### Infections and non-severe non-fatal illness overall

Similar to severe illness and death, the number of SARS-CoV-2 infections increased over the first three waves from 15.1 million in wave 1 to 20.6 million in wave 3 (table 2). Following the emergence of the Omicron variant in wave 4, the number of infections peaked in wave 4 at 34.2 million and decreased thereafter in wave 5 to 20.2 million. In the first three waves, the IFR ranged between 0.27% (95% CI 0.24% to 0.37%) and 0.59% (95% CI 0.49% to 0.69%) and was highest in the second (Beta) wave. In the fourth and fifth waves, the IFR dropped substantially to about 0.07%.

### Severe disease and death by age group

The incidence of medically and non-medically attended severe non-fatal and fatal illness for those aged 65 years and older increased noticeably from wave 1 to wave 2, and a further sharp increase for individuals 75 years and older in wave 3 (figure 2). The incidence of medically attended severe non-fatal and fatal illness in those aged 80 years and over was 802.5 (95% CI 768.6 to 835.3) and 894.1 (95% CI 815.7 to 978.2) per 100,000, respectively, in wave 3. Following the emergence of the Omicron variant in wave 4, the incidence of severe non-fatal illness and deaths decreased markedly among individuals aged 15 years and older. Among individuals aged under 15 years, the incidence of severe illness was slightly increased in the fourth wave, but decreased again in the fifth wave. Deaths among individuals aged less than 15 years remained low in all five waves. Generally, as infection waves progress, the age-specific incidence of medically attended severe non-fatal illness approaches a J-shape curve (higher at the youngest and oldest ages, but higher at the oldest ages).

### Changes in HFR and IFR with age

**Figure 3:**
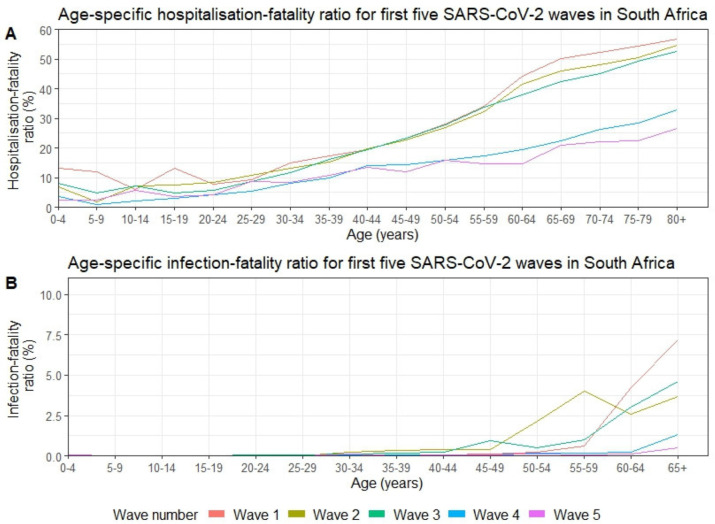
Age-specific fatality ratios (in %) for the first five SARS-CoV-2 waves in South Africa from 01 March 2020 through 13 August 2022. (A) Hospitalisation-fatality (%) and (B) infection-fatality (%).

Generally, the HFR increased with increasing age, showing a small increase in young children in some waves (figure 3, online supplemental figure 2). In wave 1, individuals aged 10–14 years had the lowest HFR of 6% (95% CI 2.3% to 10.%6) and those 80 years and older had the greatest at 57% (95% CI 51.9% to 61.4%). In individuals aged 30 years and older, there was a marked drop in HFR in the fourth and fifth waves compared with the first three waves. Individuals aged 80 years and older had the highest HFR across waves and only experienced a 53% reduction in HFR ratio from wave 1 to 5. In contrast, the HFR for individuals aged 30 years and older had a noticeably lower rate across age groups in waves 4 and 5 than in waves 1 to 3. Consider, for example, in wave 1, the difference in HFR between individuals aged 30–34 years and 80 years and above is about 42%, while in wave 5 this difference is around 18%. Like the HFR, the older age groups (65 years and above) experienced the highest IFR across all waves. For all waves, the IFR was extremely low in individuals aged <40 years.

## Discussion

We estimate the burden of SARS-CoV-2 infection and disease severity in South Africa over the first five waves of infection to understand changes in disease burden with successive waves. We found a high burden of severe illness and death in the first three waves of SARS-CoV-2, peaking in the third (Delta) wave. The emergence of the Omicron BA.1 variant was associated with very high rates of infection but substantial reductions in disease severity. Incidence of severe illness and hospitalisation fatality generally increased with increasing age. Successive waves saw a reduction in the rate of the increase in mortality with increasing age and increases in HFRs in children below 5 years of age, suggesting a shift from the epidemic state to a J-shaped distribution in mortality, typical of seasonal respiratory viruses. Notably, a high proportion of severe illness (29%) and death (55-60%) occurred outside the hospital.

The third (Delta) wave of infection had the highest incidence of severe non-fatal and fatal illness. Wave 4 (Omicron BA.1/2 variant) was characterised by marked reductions in severe disease; however, the incidence of asymptomatic, mild or moderate disease was highest in this wave. Increasing severe disease in the Beta^16^ and Delta^33^ waves has been described from South Africa and other countries, possibly related to increased virulence and relaxation of restrictions in later waves. The high attack rates and lower severity following Omicron emergence can be explained in part by the increased transmissibility and immune-evasive properties of Omicron.^14 34^ Together, these led to very high infection attack rates. The high proportion of breakthrough infections in the Omicron wave likely contributed to reduced overall severity, together with changes in virus tropism, increasing the proportion of infections in the upper airway.^35^

Successive waves of infection were associated with shifts in the age distribution of severe disease. In all five waves, severe disease was concentrated in the elderly, as has been described previously, driven in part by increasing comorbidities in this age group.^16 36 37^ Following the emergence of the Omicron variant, the reductions in severe disease were mostly seen in older adults, leading to a flattening of the steepness of increasing severity with increasing age and proportionately more severe disease in young children. This shift towards an age pattern, more typical of seasonal respiratory viruses where severe disease is concentrated in the elderly and young children, could represent the transition from a pandemic to endemic epidemiology.^11^ Notably, despite the overall reduction in disease severity, more children and adolescents were hospitalised during the Omicron wave (BA.1/2 variant) than in the previous infection waves. This pattern of proportionately more disease in children following Omicron emergence in South Africa has been described previously, however, not in the context of estimation of the burden pyramid in all age groups.^8 38 39^ Possible reasons for the shift in age distribution include changes in population immunity, pathogen virulence or changes in health-seeking behaviour or testing practices. Persons aged 60 years and older were prioritised for vaccination in South Africa as from May 2021 and extended to individuals aged 12–17 years in October 2021.^8^ This may also have contributed to the sharp decline in HFR among the elderly from wave 1 to wave 5 since vaccination coverage within South Africa reached 27% by 12 January 2022.^9^

A high proportion of severe disease and deaths occurred outside of hospitals,^12 13 40^ highlighting the importance of studies of healthcare-seeking and access and strengthening of vital registration systems to document the full burden of illness.^22^ The high number of severe cases at the peaks of the waves caused pressure on the healthcare system, potentially reducing access and exacerbating underlying health inequities.^24 41^ An important limitation of our study is that we only had healthcare utilisation data from three communities in three provinces for the first two waves of the pandemic.^24 41^ Our assumption that healthcare seeking remained constant may not be valid and could be the reason why we do not see clear trends in the SRIFR over successive waves, as we do for HFR.

Our study had other limitations. Out-of-hospital deaths were ascertained from modelling of excess COVID-19-associated deaths, using all-cause vital registration data; thus, not all excess deaths may have been due to COVID-19. Out-of-hospital deaths were ascertained from modelling of excess COVID-19-associated deaths*,* using all cause vital registration data, thus not all excess deaths may have been due to COVID-19. It is noted that the correlation between DATCOV and the excess deaths for waves 1–3 was high but decreased markedly for waves 4 and 5. No further information apart from this change in correlation was available and thus we assumed the attributable proportion was fixed for all waves. If the proportion differed from our estimate or varied over time, this could have led to bias in estimates. We are able to quantify uncertainty for most of the estimates in this paper; however, only a plausibility range is available for the proportion of excess deaths attributable to SARS-CoV-2. As a result, an important limitation of the study is the inability to fully quantify uncertainty in estimates of total and out-of-hospital deaths. The sensitivity analysis presented in the Supplement demonstrates that the magnitude of the uncertainty ranges between 1 and 9 per 100 000 population, with the lowest uncertainty observed for wave 5 and the greatest, wave 3. Data for the different strata of severity were derived from different sources. For health-seeking and infection attack rates, national data were not available, and we had to extrapolate nationally under the assumption that the available data were representative. If these measures varied by geographical region, this would have led to biased estimates. Health-seeking data were only available for the first two waves, and we assumed similar health seeking for later waves. If health-seeking changed in later waves, this could have led to over- or underestimation of out-of-hospital burden. Although the infection attack rates accounted for reinfections, the estimated hospitalisations and deaths within our study occur as they do, whether they result from reinfections or not. Within our estimation of the medically attended severe non-fatal and fatal illness, it must be noted that the proportion of in-hospital deaths attributed to SARS-CoV-2 may not necessarily have been the same as the proportion of SARS-CoV-2 hospitalisations. Finally, we used the midyear population estimates from 2020 through 2022 provided by Statistics South Africa as our population denominators throughout this study. These estimates are obtained from the Spectrum Policy Modelling system.^42^ However, the Thembisa model is also available to obtain population estimates.^3^ The total population estimated by the latter model (∼59 million)^3^ is different from the recently published Census 2022 (∼62 million)^43^ due to an underestimation of those 55 years and older.

In conclusion, we have demonstrated a high burden of infection and severe disease associated with SARS-CoV-2 over five successive waves in South Africa. With the onset of SARS-CoV-2, it was assumed that Africa would carry a large burden of infection and mortality due to weak health systems, lack of access to therapeutics and vaccines, poverty, malnutrition and high prevalence of underlying illness such as HIV and tuberculosis,^44 45^ as well as high levels of persons left undiagnosed and untreated or not effectively treated. While reports of confirmed cases suggest that Africa was relatively spared, our analysis, combining multiple data sources, suggests that this was not the case for South Africa. The estimated cumulative SARS-CoV-2 fatalities in South Africa across the first five waves (258 800-289 900) exceed the number of confirmed SARS-CoV-2 deaths of high-income countries such as Germany (150 237) and the UK (205 780).^46^ Even more stark, our estimated cumulative SARS-CoV-2 infections over this period (104.6 million) far exceed that reported for these high-income countries, namely 31.6 and 23.4 million, respectively.^46^ Furthermore, despite differences in methodology, our estimated IFR for wave 1 (0.27%) was comparable to that of Leticia, Colombia (0.28%).^47^ The discrepancy between low numbers of confirmed cases and the actual number of infections was similarly observed across six districts in Zambia. During the first wave, a SARS-CoV-2 prevalence study reported 454 708 infections compared with a mere 4917 laboratory-confirmed cases.^48^ In future, it will be important to estimate the ongoing age-specific burden of COVID-19 compared with other respiratory viral illnesses in order to decide on priorities for prevention interventions such as vaccination.

## Supplementary material

10.1136/bmjph-2024-002174 Supplementary file 1

10.1136/bmjph-2024-002174 Supplementary file 2

10.1136/bmjph-2024-002174 Supplementary file 3

10.1136/bmjph-2024-002174 Supplementary file 4

10.1136/bmjph-2024-002174 Supplementary file 5

10.1136/bmjph-2024-002174 Supplementary file 6

10.1136/bmjph-2024-002174 Supplementary file 7

10.1136/bmjph-2024-002174 Supplementary file 8

10.1136/bmjph-2024-002174 Supplementary file 9

10.1136/bmjph-2024-002174 Supplementary file 10

## Data Availability

Data may be obtained from a third party and are not publicly available.
