# Supplementary figures and images for: Burden of SARS-CoV-2 infection and severe illness in South Africa (March 2020–August 2022): a synthesis of epidemiological data
Source: BMJ Public Health. 2025 Nov 21;3(2):e002174. doi: 10.1136/bmjph-2024-002174 (PMC12645600; doi:10.1136/bmjph-2024-002174)

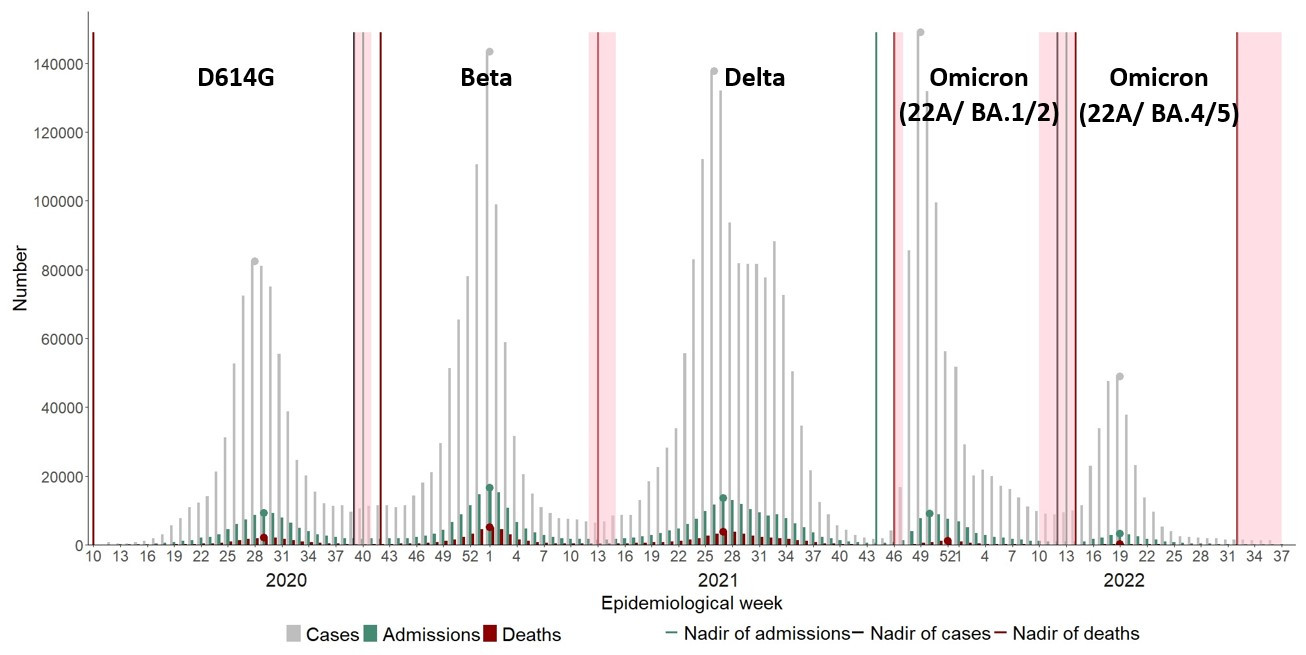

Supplement: Supplementary file 1 [file bmjph-3-2-s001.jpg]

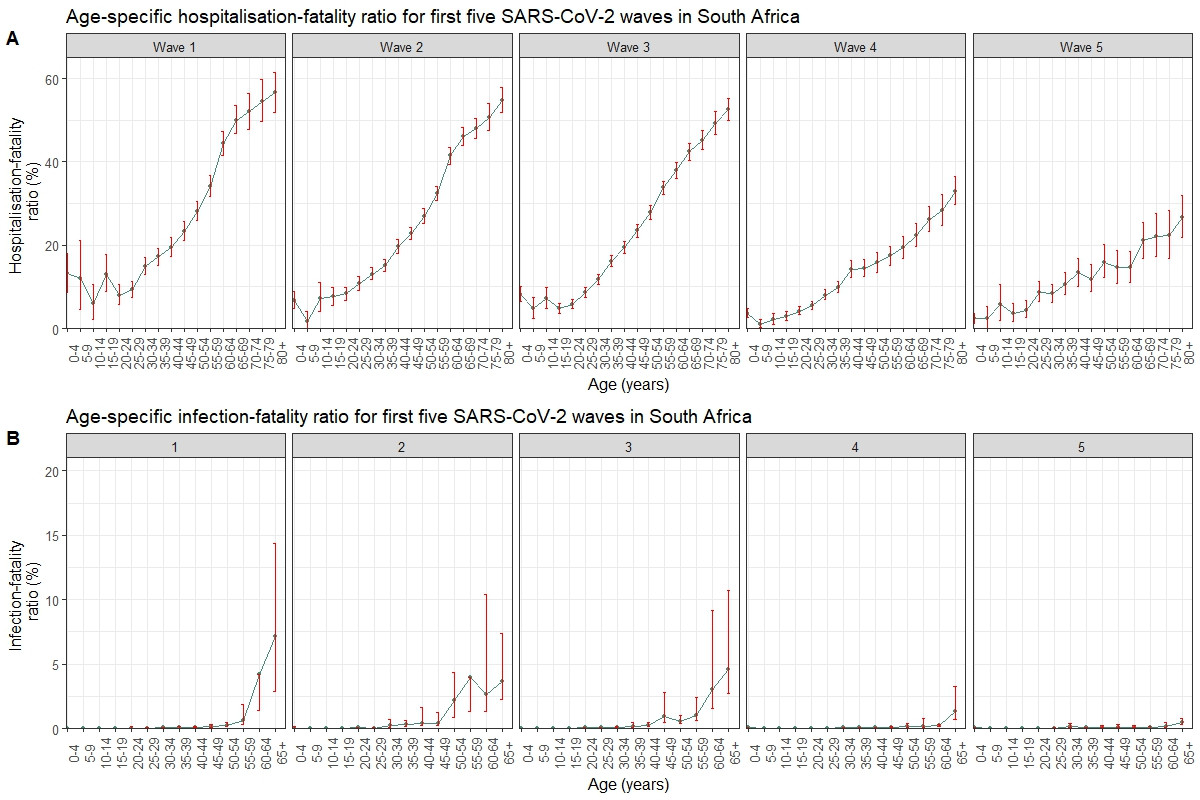

Supplement: Supplementary file 2 [file bmjph-3-2-s002.jpg]
