# Supplementary material for: Burden of SARS-CoV-2 infection and severe illness in South Africa (March 2020–August 2022): a synthesis of epidemiological data
Source: BMJ Public Health. 2025 Nov 21;3(2):e002174. doi: 10.1136/bmjph-2024-002174 (PMC12645600; doi:10.1136/bmjph-2024-002174)
Supplement: Supplementary file 3 [file bmjph-3-2-s003.docx]

**Supplement**

**S1. Wave timing definitions for the first five SARS-CoV-2 waves in South Africa**

Supplementary table 1: Wave timing definitions for the first five SARS-CoV-2 waves in South Africa

| **Wave number** | **Cases** |  |  | **Hospitalisations** |  |  | **Deaths** |  |  |
| --- | --- | --- | --- | --- | --- | --- | --- | --- | --- |
|  | **Range** |  | **Peak** | **Range** |  | **Peak** | **Range** |  | **Peak** |
|  | **Epiyear/epiweek** | **Date** | **Epiyear/ epiweek** | **Epiyear/epiweek** | **Date** | **Epiyear/ epiweek** | **Epiyear/epiweek** | **Date** | **Epiyear/ epiweek** |
| **1** | 2020/10–2020/39 | 1 March 2020–6 September 2020 | 2020/28 | 2020/10–2020/40 | 1 March 2020–3 October 2020 | 2020/29 | 2020/10–2020/42 | 01 March 2020 – 17 October 2020 | 2020/29 |
| **2** | 2020/40–2021/13 | 27 September 2020–03 April 2021 | 2021/01 | 2020/41–2021/13 | 4 October 2020–3 April 2021 | 2021/01 | 2020/43–2021/13 | 18 October 2020 – 03 April 2021 | 2021/01 |
| **3** | 2021/14–2021/44 | 4 April 2021–06 November 2021 | 2021/26 | 2021/14–2021/44 | 4 April 2021–6 November 2021 | 2021/27 | 2021/14–2021/46 | 04 April 2021 – 20 November 2021 | 2021/27 |
| **4** | 2021/45–2022/12 | 7 November 2021–26 March 2022 | 2021/49 | 2021/45–2022/13 | 7 November 2021–2 April 2022 | 2021/50 | 2021/47–2022/14 | 21 November 2021 – 09 April 2022 | 2021/52 |
| **5** | 2022/13–2022/32 | 27 March 2022–13 August 2022 | 2022/19 | 2022/14–2022/32 | 3 April 2022–13 August 2022 | 2022/19 | 2022/15–2022/32 | 10 April 2022 – 13 August 2022 | 2022/19 |

**S2. Detailed description of burden estimation approach**

***Severe non-fatal and fatal illness***

1. *Medically attended severe non-fatal illness (incidence of hospitalisation)*

Using the SARS-CoV-2 hospitalisation wave definitions, all hospital admissions (B), hospital admissions with admission reasons known (C), and hospital admissions explicitly attributed to SARS-CoV-2 (A) were extracted from DATCOV, respectively. The proportion of hospital admissions explicitly attributed to SARS-CoV-2 among all admissions with known admission reasons was defined as:

$\frac{A}{C}$ (S1)

The total in-hospital deaths (D) were determined from DATCOV using the SARS-CoV-2 in-hospital deaths definitions. The adjusted number of severe, medically attended SARS-CoV-2 hospitalisations was obtained by subtracting the in-hospital deaths (D) from the total number of admissions (B) and multiplying the outcome by the proportion of explicitly attributed SARS-CoV-2 admissions (from (1)). That is,

$\left( B-D \right)\times\frac{A}{C}$ (S2)

Finally, the medically attended severe non-fatal illness incidence rate per 100,000 ($S_{M})$ was calculated by dividing the adjusted number of SARS-CoV-2 hospitalisations (from (2)) by the respective national population estimate at the peak of the SARS-CoV-2 hospitalisations wave (P_H_) and multiplying by 100,000:

$S_{M}=\left[ \left( B-D \right)\times\frac{A}{C} \right]\times\frac{100000}{P_{H}}$ (S3)

1. *Medically attended fatal illness (incidence of in-hospital death)*

The in-hospital explicitly attributed SARS-CoV-2 deaths from explicitly attributed SARS-CoV-2 hospital admissions (E) were determined from DATCOV using the SARS-CoV-2 in-hospital deaths wave definitions. The hospitalisation-fatality ratio (HFR) (defined as the proportion of SARS-CoV-2 deaths among SARS-CoV-2 hospitalised individuals)^1^ was determined as follows:

$\frac{E}{A}$ (S4)

The adjusted number of total hospitalisations was then calculated by dividing the adjusted number of SARS-CoV-2 hospitalisations (from (2)) by the proportion of hospitalisations that did not result in death, i.e., 1 – HFR (from (4)). That is,

$\frac{\left[ \left( B-D \right)\times\frac{A}{C} \right]}{\left[ 1- \frac{E}{A} \right]}$ (S5)

The medically attended fatal illness incidence rate per 100,000 ($F_{M})$ was then determined by subtracting from (5) the adjusted SARS-CoV-2 hospitalisations (from (2)), dividing by the respective national population estimate at the peak of the SARS-CoV-2 in-hospital deaths wave ($P_{D})$, and multiplying by 100,000:

$F_{M}= \left( \left\{ \frac{\left[ \left( B-D \right)\times\frac{A}{C} \right]}{\left[ 1- \frac{E}{A} \right]} \right\}- \left[ \left( B-D \right)\times\frac{A}{C} \right] \right) \times\frac{100000}{P_{D}}$ (S6)

1. *Non-medically attended severe non-fatal illness*

The incidence of non-medically attended severe non-fatal illness ($S_{N})$ is defined as the difference between the incidence of hospitalisation ($S_{M})$ (from (3)) divided by the average proportion of severe illness seeking care from the HUS (J) and the incidence of hospitalisation ($S_{M})$, i.e.,

$S_{N}=$ $\frac{S_{M}}{J}- S_{M}$ (S7)

We assumed the average proportion of severe illness seeking care from the HUS remains unchanged irrespective of age strata or wave as the survey did not have sufficient power to stratify, and estimates were not available by wave.

1. *Non-medically attended fatal illness (incidence of out-of-hospital deaths)*

We assumed that 85% of excess deaths (G) were attributable to SARS-CoV-2 based on published data.^2^ The incidence of out-of-hospital deaths per 100,000 ($F_{N})$ was then calculated as the SARS-CoV-2 attributable excess deaths ($0.85G)$less the adjusted SARS-CoV-2 in-hospital deaths, divided by the respective national population estimates at the peak of the SARS-CoV-2 deaths waves ($P_{D})$ times 100,000. That is,

$F_{N}= \left( 0.85G- \left( \left\{ \frac{\left[ \left( B-D \right)\times\frac{A}{C} \right]}{\left[ 1- \frac{E}{A} \right]} \right\}- \left[ \left( B-D \right)\times\frac{A}{C} \right] \right) \right) \times\frac{100000}{P_{D}}$ (S8)

For the age-specific estimates, age groups for which in-hospital deaths exceeded the attributable excess deaths, the non-medically attended fatal illness estimate was set to zero, i.e., the SARS-CoV-2 attributable excess deaths were assumed equal to the in-hospital deaths. Since we are unable to quantify the uncertainty associated with the proportion of total deaths attributable to SARS-CoV-2 we conducted an additional sensitivity analysis (refer to Appendix S3). Herein we considered varying percentages of excess deaths attributable to SARS-CoV-2 within the range specified by published data, that is 85-95%.^2^

***Non-severe, non-fatal illness (i.e., moderate, mild and asymptomatic illness)***

The non-severe, non-fatal infection incidence (I) was obtained by multiplying the wave and age-specific attack rates (H) from PHIRST-C (refer to Section 1.2 (d)) by 100,000 and subtracting out the medically and non-medically attended severe non-fatal and fatal illness (from (3),(6) – (8)) since disease severity strata are assumed mutually exclusive:

$I= 100000H-\left( F_{N}+ F_{M} \right)-(S_{N}+ S_{M})$ (S9)

**S3. Sensitivity analysis: Excess deaths attributable to SARS-CoV-2**

Supplementary table 2: Wave-specific incidence of non-medically attended fatal SARS-CoV-2 infections in South Africa per 100 000 population at risk from 01 March 2020 through 13 August 2022 under assumption of varying proportion of excess deaths attributable to SARS-CoV-2.

| **Wave number/**  **Dominant variant** | **Non-medically attended fatal**  ***Incidence/100 000 population*** | | | **Total fatal**  ***Incidence/100 000 population*** | | | **Non severe non-fatal and non-fatal^b^**  ***Incidence/100 000 population***  **(95% CI)** | | |
| --- | --- | --- | --- | --- | --- | --- | --- | --- | --- |
|  | **Excess death proportion** | | | **Excess death proportion** | | | **Excess death proportion** | | |
|  | **85%** | **90%** | **95%** | **85%** | **90%** | **95%** | **85%** | **90%** | **95%** |
| **1/**  **D614G** | 20.9 | 24.9 | 28.9 | 68.2 | 72.2 | 76.2 | 25 215.1  (18 421.8, 27 884.6) | 25 211.1  (18 417.7, 27 880.6) | 25 207.1  (18 413.7,27 876.6) |
| **2/**  **Beta** | 81.7 | 90.1 | 98.5 | 143.0 | 151.4 | 159.8 | 23 829.5  (20 516.9, 29 106.6) | 23 821.1  (20 508.5, 29 098.2) | 23 812.7  (20 500.1,29 089.8) |
| **3/**  **Delta** | 87.1 | 96.2 | 105.4 | 156.2 | 165.4 | 174.6 | 33 991.5  (28 477.3, 37 441.5) | 33 982.3  (28 468.1, 37 432.3) | 33 973.1  (28 458.9,37 423.1) |
| **4/**  **Omicron BA.1/2** | 27.3 | 29.6 | 32.0 | 39.5 | 41.9 | 44.2 | 56 612.3  (53 114.7, 62 679.4) | 56 610.0  (53 112.4, 62 677.0) | 56 607.7  (53 110.1,62 674.7) |
| **5/**  **Omicron BA.4/5** | 20.2 | 21.7 | 23.1 | 24.4 | 25.8 | 27.2 | 33 324.7  (31 033.6, 40 980.1) | 33 323.3  (31 280.9, 40 493.4) | 33 321.9  (31 030.8,40 977.2) |

1. Epidemic and Pandemic Preparedness and Prevention (EPP), WHO Headquarters (HQ), WHO Worldwide. *Estimating Mortality from COVID-19*. World Health Organisation (WHO); 2020:1-4. Accessed September 19, 2023. https://www.who.int/publications/i/item/WHO-2019-nCoV-Sci-Brief-Mortality-2020.1

2. Moultrie T, Dorrington R, Laubscher R, Groenewald P, Bradshaw D. *Correlation of Excess Natural Deaths with Other Measures of the COVID-19 Pandemic in South Africa*. Burden of Disease Research Unit South African Medical Research Council; 2021:1-14. Accessed May 20, 2024. https://www.samrc.ac.za/sites/default/files/bod/weeklyreports/CorrelationExcessDeaths.pdf
